# Hypertension Subtypes among Thai Hypertensives: An Analysis of Telehealth-Assisted Instrument in Home Blood Pressure Monitoring Nationwide Pilot Project

**DOI:** 10.1155/2020/3261408

**Published:** 2020-04-09

**Authors:** Sakolwat Montrivade, Pairoj Chattranukulchai, Sarawut Siwamogsatham, Yongkasem Vorasettakarnkij, Witthawat Naeowong, Patchaya Boonchayaanant, Anut Sakulsupsiri, Aekarach Ariyachaipanich, Vorarit Lertsuwunseri, Voravut Rungpradubvong, Sudarat Satitthummanid, Sarinya Puwanant, Somchai Prechawat, Suphot Srimahachota, Jarkarpun Chaipromprasit, Wacin Buddhari, Smonporn Boonyaratavej, Surapun Sitthisook, Peera Buranakitjaroen, Apichard Sukonthasarn, Somkiat Sangwatanaroj

**Affiliations:** ^1^Division of Cardiovascular Medicine, Chulalongkorn University, King Chulalongkorn Memorial Hospital, 10330 Bangkok, Thailand; ^2^Division of Hospital and Ambulatory Medicine, Chulalongkorn University, King Chulalongkorn Memorial Hospital, 10330 Bangkok, Thailand; ^3^Division of Endocrinology and Metabolism, Department of Medicine, Faculty of Medicine, Chulalongkorn University, King Chulalongkorn Memorial Hospital, 10330 Bangkok, Thailand; ^4^Pharmacy Department, King Chulalongkorn Memorial Hospital, 10330 Bangkok, Thailand; ^5^Department of Medicine, Siriraj Hospital, Mahidol University, 10700 Bangkok, Thailand; ^6^Thai Hypertension Society, 10310 Bangkok, Thailand

## Abstract

**Background:**

White-coat hypertension (HT), masked HT, HT with white-coat effect, and masked uncontrolled HT are well-recognized problems of over- and undertreatment of high blood pressure in real-life practice. However, little is known about the true prevalence in Thailand.

**Objectives:**

To examine the prevalence and characteristics of each HT subtype defined by mean home blood pressure (HBP) and clinic blood pressure (CBP) using telemonitoring technology in Thai hypertensives.

**Methods:**

A multicenter, observational study included adult hypertensives who had been diagnosed for at least 3 months based on CBP without the adoption of HBP monitoring. All patients were instructed to manually measure their HBP twice a day for the duration of at least one week using the same validated automated, oscillometric telemonitoring devices (Uright model TD-3128, TaiDoc Corporation, Taiwan). The HBP, CBP, and baseline demographic data were recorded on the web-based system. HT subtypes were classified according to the treatment status, CBP (≥or <140/90 mmHg), and mean HBP (≥or <135/85 mmHg) into the following eight subtypes: in nonmedicated hypertensives, there are four subtypes that are normotension, white-coat HT, masked HT, and sustained HT; in treated hypertensives, there are four subtypes that are well-controlled HT, HT with white-coat effect, masked uncontrolled HT, and sustained HT.

**Results:**

Of the 1,184 patients (mean age 58 ± 12.7 years, 59% women) from 46 hospitals, 1,040 (87.8%) were taking antihypertensive agents. The majority of them were enrolled from primary care hospitals (81%). In the nonmedicated group, the prevalence of white-coat and masked HT was 25.7% and 7.0%, respectively. Among the treated patients, the HT with white-coat effect was found in 23.3% while 46.7% had uncontrolled HBP (a combination of the masked uncontrolled HT (9.6%) and sustained HT (37.1%)). In the medicated older subgroup (*n* = 487), uncontrolled HBP was more prevalent in male than in female (53.6% vs. 42.4%, *p*=0.013).

**Conclusions:**

This is the first nationwide study in Thailand to examine the prevalence of HT subtypes. Almost one-fourth had white-coat HT or HT with white-coat effect. Approximately half of the treated patients especially in the older males had uncontrolled HBP requiring more intensive interventions. These results emphasize the role of HBP monitoring for appropriate HT diagnosis and management. The cost-effectiveness of utilizing THAI HBPM in routine practice needs to be examined in the future study.

## 1. Introduction

Hypertensive patients can be divided into several subtypes based on clinic blood pressure (CBP) and out-of-office blood pressure values including white-coat hypertension (HT), masked HT and sustained HT in nonmedicated patients or HT with white-coat effect, masked uncontrolled HT, and sustained HT in patients receiving antihypertensive medications [1–3]. However, the diagnosis is very challenging and is often overlooked since it requires both CBP and out-of-office BP data [4]. Previous studies reported the prevalence of white-coat HT and masked HT as high as 35% and 10%, respectively [2, 3, 5–7]. In patients with white-coat HT and HT with white-coat effect, the overintensification of antihypertensive medications could potentially cause hypotension and worsen cardiovascular outcomes especially in the elderly [8, 9]. On the other hand, patients with masked HT and masked uncontrolled HT may be at an increased risk of stroke comparable to those in patients with sustained HT [10]. Out-of-office BP measurement is crucial to confirm the diagnosis and to titrate BP-lowering medications in the patient with these HT subtypes [11, 12]. Home blood pressure monitoring (HBPM) is recommended by recent several guidelines [13–15] as a practical modality to obtain out-of-office BP, which is less expensive, less complex, and more widely available than ambulatory blood pressure monitoring (ABPM) [16–19]. The adoption of telemonitoring strategy, an Internet-based transmission system, allows linking home blood pressure (HBP) records between multiple HBPM devices and a central computer at the clinic. The data can be monitored and analyzed by trained healthcare professionals remotely and can facilitate improvement in managing hypertensive patients [11, 20–22]. This technology overcomes the self-reporting bias which is a limitation of HBPM in a clinical practice [23] since the HBP data transferring is completed without manual data entry by the patient.

The telehealth-assisted instrument in home blood pressure monitoring (THAI HBPM) project was designed to be a proof-of-concept observational multicenter study implementing the web-based telemonitoring. We aimed to examine the prevalence and characteristics of HT subtypes defined by mean HBP and CBP in real-life clinical setting across Thailand.

## 2. Materials and Methods

### 2.1. Study Oversight

THAI HBPM is a nationwide prospective observational study involving 46 centers across all regions of Thailand (*see Supplementary Material for the details of all participating sites*). The Ministry of Public Health of Thailand promoted the nationwide project and approved the study protocol and the centralized institutional review board review process.

### 2.2. Patient Population

Eligible participants were consecutively enrolled from 46 centers throughout the country. Adult patients who were 18 years of age or older with known HT diagnosed for more than 3 months based on CBP without adoption of HBPM were enrolled. If the participants were taking antihypertensive agents, they must have been on a stable dose of medications for at least 3 months before the enrollment. Patients with incomplete clinical characteristics or BP data will be excluded.

### 2.3. Clinic and Home Blood Pressure Measurement

Clinic BP was measured by trained healthcare professionals using the validated sphygmomanometer, after the patient had been resting in a relaxed, seated position [24, 25]. We used the average of two consecutive readings at a 2-minute interval taken from the arm with the higher BP for the analysis.

Clinical validation between CBP and HBP readings was performed at the clinic according to the standard recommendation [26] before starting the HBP recording (day 0). The device is validated if there are less than 5 mmHg differences of both SBP and DBP between sphygmomanometers and HBPM devices [26, 27] (see Supplementary [Supplementary-material supplementary-material-1]).

**Figure 1:**
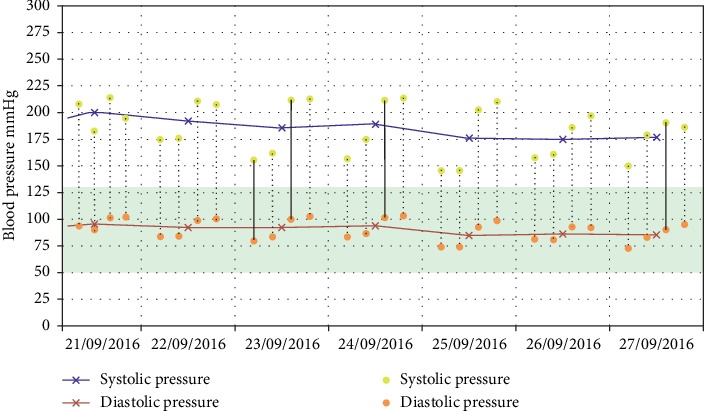
Example of consecutive 7-day home blood pressure (HBP) data viewed on the desktop computer via Uright Telehealth website of a 55-year-old woman with a 2-year history of HT. The mean HBP was 184/94 mmHg while her clinic BP was 185/90 mmHg; hence, this BP pattern was categorized as sustained HT. Purple crosses represent daily average systolic HBP. Red crosses represent daily average diastolic HBP.

Home BP data were obtained using the same validated automated, oscillometric devices (Uright model TD-3128, TaiDoc Technology Corporation, Taiwan, see Supplementary [Supplementary-material supplementary-material-1]). Trained healthcare providers instructed the participants to self-record HBP twice a day (1 hour after waking in the morning before taking antihypertensive medications or having breakfast and 30 minutes before going to bed) after 3 minutes of rest in a sitting position with two consecutive measurements, 1 minute apart for each recording. Blood pressure measurement continued for at least 7 days as recommended by standard guidelines [2, 28] during a 30-day period. In case there is a significantly different BP between both arms as determined at the enrollment visit, participants were instructed to use the arm with the greater BP. To avoid self-reporting bias, HBP values were automatically recorded in device memory. All participants were informed to bring the HBPM device along with them on the appointed clinic visit. At the 1^st^ follow-up visit (day 30–45), all recorded HBP data were transferred from the devices via USB cable to the Windows-based computer at the participating clinics. The data will then be automatically forwarded to cloud storage through the Internet-based transmission system. When HBP data had been uploaded, they could be viewed and analyzed using a regular Internet Explorer program via Uright Telehealth website (Figure 1). The device and Uright telehealth system were validated and approved by the US FDA [29]. A trained investigator at King Chulalongkorn Memorial Hospital who was blinded to the study demographic data independently interpreted the BP pattern. Patients who had at least 7-day HBP records will be included in the analysis. We discarded the measurements taken on the first day and used the mean value of all the remaining HBP records for the data analysis [16]. The CBP, demographic data, medical history, biochemistry laboratory results, and current antihypertensive medications were recorded on the web-based system.

### 2.4. Data Analysis

Participants were categorized according to the treatment status, CBP (≥or <140/90 mmHg), and HBP data (≥or <135/85 mmHg) into the following 8 subtypes [2, 28, 30]. In nonmedicated patients, there were 4 subtypes: (1) normotension: nonhypertensive CBP and HBP levels; (2) white-coat HT: hypertensive CBP level and nonhypertensive HBP level; (3) masked HT: nonhypertensive CBP level and hypertensive HBP level; and (4) sustained HT: both hypertensive CBP and HBP levels. Treated participants were categorized into another 4 subtypes: (5) well-controlled HT: nonhypertensive CBP and HBP levels; (6) HT with white-coat effect: hypertensive CBP level and nonhypertensive HBP level; (7) masked uncontrolled HT: nonhypertensive CBP level and hypertensive HBP level; and (8) sustained HT: both hypertensive CBP and HBP levels.

Regarding the HBP control status in treated patients, the “controlled HBP group” consists of patients with well-controlled HT and HT with white-coat effect while the “uncontrolled HBP group” includes 2 other subtypes: masked uncontrolled HT and sustained HT. The white-coat effect (CBP and HBP difference) was calculated by mean CBP minus mean HBP (mmHg). Subgroup analyses were analyzed according to the country's regions, gender, and age (<60 years and ≥60 years).

Categorical variables were described as numbers (*n*) and percentage of frequencies (%). Continuous variables were shown as mean values and SD. Chi-square test and ANOVA were used for the analysis of categorical and continuous variables, respectively. We used SPSS software, version 22.0 (IBM) for statistical analysis.

## 3. Results

### 3.1. Patient Characteristics

A total of 1,250 patients were consecutively enrolled between August 2016 and August 2017. Of these, 66 were excluded due to incomplete clinical characteristics or BP data. Thus, 1,184 patients from 46 hospitals (5 regions: North, Northeast, East, Center, and South) were included in the analysis (see patient enrollment flow chart in Supplementary [Supplementary-material supplementary-material-1]). Patient characteristics are summarized in Table 1. The mean (±SD) age of the patients was 58.2 ± 12.7 years; 59% were women. The majority of them were recruited from primary care hospitals (81%). The mean duration of the diagnosis of HT was 8.4 ± 3.1 months. There were 1,040 (87.8%) patients on antihypertensive medications. Most of them took one or two agents per day (39.0% and 36.1%, resp.). The most commonly used medications were dihydropyridine calcium channel blockers (62.3%), followed by angiotensin converting enzyme (ACE) inhibitors (45.6%), while the diuretics were used in 18.6%. The mean (±SD) BMI in the cohort was 26.5 ± 5.1 kg/m^2^. The mean (±SD) clinic and home BP were 143.1 ± 18.0/84.8 ± 11.7 mmHg and 134.3 ± 13.9/80.6 ± 8.8 mmHg, respectively, with the overall white-coat effect (systolic BP/diastolic BP difference between CBP and HBP) of 8.9 ± 16.4/4.2 ± 9.8 mmHg.

There was no significant difference between nonmedicated and treated patients in all BP components except mean clinic diastolic BP and mean home diastolic BP which were higher in the nonmedicated group (89.7 ± 11.1 vs. 84.1 ± 11.7 mmHg, *p* < 0.001, and 82.6 ± 8.7 vs. 80.4 ± 8.8 mmHg, *p*=0.004, resp.).

### 3.2. Hypertension Subtypes

The prevalence of all 8 HT subtypes is presented in Table 2. Of 144 nonmedicated patients, the prevalence of white-coat HT was 25.7% with the white-coat effect of 21.2 ± 4.6/10.2 ± 4.1 mmHg and the prevalence of masked HT was 7.0%. There were 83 patients with sustained HT (57.6%). Approximately 10% of nonmedicated participants were found to have normotension since they had normal repeated CBP and mean HBP data. Of 1,040 treated participants, the proportion of patients who had HT with white-coat effect was 23.3% (white-coat effect of 24.5 ± 5.7/10.1 ± 6.6 mmHg). Well-controlled HT, masked uncontrolled HT, and sustained HT were prevalent in 30.0%, 9.6%, and 37.1% of the treated group, respectively. A total of 486 treated patients (46.7%) had uncontrolled HBP (masked uncontrolled HT and sustained HT). There were no differences in baseline characteristics (age, gender, comorbid diseases, and laboratory results) between patients in each HT subtype as summarized in Tables 3 and 4.

### 3.3. HT Subtypes by Age and Gender

HT subtypes stratified according to age and gender (≤60 years and >60 years) is shown in Table 5. In the treated older (>60 years old) subgroups (*n* = 499), uncontrolled HBP was more prevalent in male than female (53.7% vs. 42.4%, *p*=0.013) but there was no significant difference between genders in younger subgroups (42.5% and 48.3%, *p*=0.188). The older males had higher prevalence of uncontrolled HBP than younger males (*p*=0.02) without significant difference in female groups (*p*=0.151).

### 3.4. HT Subtype Analyzed by Hospital Regions

Table 6 shows the HT subtype categorized by hospital regions. Most of the patients were enrolled from the Northeast region (*n* = 276, 23.3%) followed by the Central region (*n* = 252, 21.3%). The prevalence of HT subtypes was not significantly different according to the hospital regions. Of all five regions, the East region tended to have the highest rate of uncontrolled HBP (53.3%) followed by the South (48.8%) without significant difference when compared with the rest of the country (*p*=0.317).

## 4. Discussion

This is the first nationwide multicenter study to examine the prevalence of HT subtypes in Thai hypertensives using telemonitoring. We assessed patient characteristics and type of antihypertensive medications and further analyzed subgroup of patients according to gender, age, and geographical regions. In nonmedicated group of the present study, the proportion of patients with white-coat HT and masked HT was 25.7% and 7.0%, respectively, which was concordant with several published studies. Piper et al. [1] recently conducted a systematic review including studies using HBPM and found a wide range of prevalence of white-coat HT from 16% to 55%. Stergiou et al. [31] reported the International Database of Home blood pressure in relation to Cardiovascular Outcomes (IDHOCO) study involving 6,458 participants from 5 different populations. They found that 9.8% of the participants had masked HT. In comparison with the studies using ABPM, Omboni et al. [5] included 14,143 patients from 27 countries and reported the prevalence of white-coat HT and masked HT of 22.6% and 11.1%, respectively. In the IDACO ABPM registry, white-coat HT was found in 35.7% while the prevalence of masked HT was 16.9% [6]. One possibility of a lower proportion of masked HT in our study is that we defined masked HT using the mean morning and evening HBP values. In contrast with ABPM method, we could not identify the elevated midday BP and high nocturnal BP during sleep, which are common phenotypes of masked HT [10, 32].

In treated hypertensives, the proportion of patients with HT with white-coat effect and masked uncontrolled HT in the present cohort was 23.3% and 9.6%, respectively. These numbers are quite similar to 23% and 9% reported from the recent Asia BP@Home study that included the patients from 11 countries across Asia [33]. Comparing with the western study, Stergiou [34, 35] et al. reported the prevalence of HT with white-coat effect and masked uncontrolled HT of 22% and 11.9%, respectively, by using average 2-visit CBP value and 4-day HBPM value. We further investigated the white-coat effect across the cohort and found the effect of 8.7 ± 16.9/3.8 ± 10.0 mmHg in patients receiving antihypertensive medications, while the effect was 10.2 ± 12.9/7.2 ± 7.8 mmHg in the nonmedicated patients. These ranges of white-coat effect found in our study are comparable to the previous reports using HBPM [36, 37].

In the present study, the controlled HBP was achieved in 53.3% of treated patients, which is comparable to 51% from the cross-sectional survey over 25 provinces across Thailand in 2011 [38]. However, this rate is lower than the result from the 2014 Thai National Health Examination Survey V showing that 60% of hypertensive participants had controlled BP using field BP target of <140/90 mmHg taken by the community health volunteer home visit [39]. The differences in the patient demographic and BP measurement method could account for the higher BP control rate.

Subgroup analysis of patients according to sex and age found that older males had the least HBP controlled (only 46.4%), whereas the older females had the most (57.6%). A recent randomized controlled trial conducted in Thai primary care setting showed that HBPM in older patients significantly decreased the rate of uncontrolled HT from 90% to 38.2% in one year, compared with usual care (from 81.8% to 54.5%) [40]. These findings could emphasize the role of HBPM for long-term BP control in the older population, especially in the males.

Regarding the BP control of each geographical region across Thailand, the East region tended to have the greatest rate of uncontrolled HBP whereas the Northeast tended to have the lowest (57.2% and 46.7%). This interregional difference may be attributed to the fact that the Northeast region has the lowest prevalence of HT compared with other regions [39], the difference in healthcare systems, and high salt intake in the East region. More importantly, this data should prompt local healthcare authorities for further evaluation and action to improve HT care.

Telehealth technology has been strongly recommended in the recent guidelines for the prevention, detection, and management of high BP in adults [11, 15]. It can be implemented with adjunct active interventions from healthcare providers such as the titration of medication or giving feedback to the patients or it can be used as only passive telemonitoring [41, 42] as demonstrated in our study. A recent meta-analysis of randomized controlled studies showed that the effect of home telemonitoring on BP control was greater than that of BP self-monitoring without transmission of HBP data [21]. This emphasizes an incremental value of the teletransmission approach to minimize self-reporting bias [23]. The present study shows that implementing telehealth-assisted HBPM technology in Thailand was feasible. We constructed a network of hypertensive care across all regions and provided Internet-based online database. Achieving target BP control required monitoring and an excellent standard of care. Thus, our study could be the first step to enhance the role of technological advances for BP control in Thailand. The ongoing 1-year follow-up study of THAI HBPM to examine the BP control rate after implementing the HBPM-facilitated medication titration could further highlight the role of telemonitoring in the management of HT.

The present study has some limitations. Firstly, the majority of the participants were enrolled from primary care hospitals and managed by general practitioners; thus the results may not be generalizable to the larger scale hospitals. However, our finding can still represent the characteristic of HT subtypes in real-life practice since the vast majority of Thai hypertensives have been followed at primary care centers [40]. Secondly, the proportion of patients with white-coat and masked HT may not be accurate since the number of patients in the nonmedicated group was small. Moreover, this study enrolled the participants solely based on high CBP; thus the majority of patients with masked HT who have normal or borderline CBP may have not been included. Thirdly, since the data on current smoking status is not available, this could affect the characteristic of masked HT. The out-of-clinic smoking potentially affects the raising of BP at home [32]. Lastly, due to the fact that this study was conducted in various clinics across the country, thus CBP data were obtained from different sphygmomanometers' models. However, we minimized intraobserver and interobserver variations by training the staff to perform CBP and clinical validation in the same manner according to standard guideline recommendations [2, 26, 27]. It is noteworthy that the strength of our study is that we used a single model of validated HBP device for all study sites, which resulted in highly robust HBP data. Moreover, we implemented the cloud-based transmission of HBP data that overcomes the self-reporting bias, which is a limitation of conventional HBPM in real-world practice.

## 5. Conclusions

This is the first nationwide study in Thailand to demonstrate the prevalence and characteristics of HT subtypes in Thai hypertensives using telemonitoring. Almost one-fourth had white-coat HT or HT with white-coat effect. Approximately half of the treated hypertensives, especially in the older males, had uncontrolled HBP which requires more intensive interventions. The cost-effectiveness of utilizing THAI HBPM for long-term BP control in routine practice needs to be examined in the future study.

## Figures and Tables

**Table 1:** Demographics and clinical characteristics of the patient population (*n* = 1,184). Values are number (%) or mean ± SD.

Characteristic	Value
Age (years)	58.2 ± 12.7
Female	695 (58.7%)
Diabetes	158 (13.3%)
Dyslipidemia	610 (51.5%)
Follow-up at primary care hospital	960 (81.0%)
On antihypertensive therapy	1,040 (87.8%)
Number of antihypertensive medications	
1	406 (39.0%)
2	375 (36.1%)
≥3	259 (24.9%)
Type of antihypertensive medications	
Dihydropyridine calcium channel blockers	648 (62.3%)
Angiotensin converting enzyme inhibitors	474 (45.6%)
Diuretics	193 (18.6%)
Others	636 (61.2%)
BMI (kg/m^2^)	26.5 ± 5.1
Clinic BP	
Systolic BP (mmHg)	143.1 ± 18.0
Diastolic BP (mmHg)	84.8 ± 11.7
Pulse (beats/min)	79.2 ± 12.2
Home BP	
Systolic BP (mmHg)	134.3 ± 13.9
Diastolic BP (mmHg)	80.6 ± 8.8
Pulse (beats/min)	74.6 ± 9.9
Laboratory results	
Total cholesterol (mg/dL)	199.2 ± 41.2
Triglyceride (mg/dL)	152.3 ± 85.5
HDL (mg/dL)	53.4 ± 14.7
Calculated LDL (mg/dL)	115.0 ± 38.5
Creatinine (mg/dL)	0.94 ± 0.56
GFR <60 mL/min/1.73 m^2^	268 (22.6%)

BMI; body mass index, BP; blood pressure, HDL; high-density lipoprotein, LDL; low-density lipoprotein, GFR; glomerular filtration rate.

**Table 2:** Prevalence of eight hypertension (HT) subtypes categorized according to the treatment status, clinic blood pressure, and mean home blood pressure.

Nonmedicated patients (*n* = 144)		Home blood pressure	
		SBP <135 and DBP <85 mmHg	SBP ≥135 and/or DBP ≥85 mmHg

Clinic blood pressure	SBP <140 and DBP <90 mmHg	Normotension 14 (9.7%)	Masked HT 10 (7.0%)
	SBP ≥140 and/or DBP ≥90 mmHg	White-coat HT 37 (25.7%)	Sustained HT 83 (57.6%)

Treated patients (*n* = 1,040)		Home blood pressure	
		SBP <135 and DBP <85 mmHg	SBP ≥135 and/or DBP ≥85 mmHg

Clinic blood pressure	SBP <140 and DBP <90 mmHg	Well-controlled HT 312 (30.0%)	Masked uncontrolled HT 100 (9.6%)
	SBP ≥140 and/or DBP ≥90 mmHg	HT with white-coat effect 242 (23.3%)	Sustained HT 386 (37.1%)

SBP; systolic blood pressure, DBP; diastolic blood pressure.

**Table 3:** Patient characteristics categorized by hypertension subtypes in the nonmedicated group.

Characteristic	All (*n* = 144)	Normotension (*n* = 14)	White-coat HT (*n* = 37)	Masked HT (*n* = 10)	Sustained HT (*n* = 83)	*p* value
Age (years)	53.8 ± 12.1	51.3 ± 11.4	53.3 ± 10.5	54.2 ± 12.6	53.5 ± 13.1	0.262
Female	77 (53.5%)	7 (50%)	22 (59.5%)	6 (60.0%)	42 (50.6%)	0.623
Diabetes	1 (0.7%)	—	1 (2.7%)	—	—	—
Dyslipidemia	52 (36.1%)	4 (28.6%)	12 (32.4%)	5 (50%)	25 (30.1%)	0.351
BMI (kg/m^2^)	25.4 ± 4.4	25.1 ± 4.2	25.3 ± 5.4	25.3 ± 3.2	25.7 ± 4.0	0.911
Clinic BP						
Systolic BP (mmHg)	143.2 ± 16.8	122.4 ± 7.6	148.4 ± 8.0	129.4 ± 7.9	154.3 ± 12.4	**<0.001**
Diastolic BP (mmHg)	89.7 ± 11.1	74.8 ± 7.2	90.1 ± 6.8	79.5 ± 6.3	97.1 ± 9.3	**<0.001**
Pulse (beats/min)	79.2 ± 11.8	76.1 ± 8.5	78.8 ± 10.0	78.7 ± 5.4	80.9 ± 11.2	0.531
Home BP						
Systolic BP (mmHg)	133.0 ± 12.0	120.2 ± 8.1	127.3 ± 5.5	146.6 ± 6.4	142.0 ± 8.7	0.435
Diastolic BP (mmHg)	82.6 ± 8.7	72.4 ± 6.6	77.9 ± 4.6	84.4 ± 5.3	89.3 ± 6.3	**<0.001**
Pulse (beats/min)	75.9 ± 8.7	74.1 ± 8.7	76.0 ± 7.7	77.7 ± 7.4	77.0 ± 9.1	0.334
Laboratory results						
Total cholesterol (mg/dL)	210.4 ± 50.0	221.6 ± 68.1	211.4 ± 27.8	212.7 ± 58.0	202.0 ± 42.1	0.713
Triglyceride (mg/dL)	134.0 ± 74.9	132.0 ± 73.0	103.3 ± 40.5	145.6 ± 65.0	134.9 ± 88.3	0.382
HDL (mg/dL)	54.8 ± 13.7	53.4 ± 11.3	59. 5 ± 10.0	51.1 ± 8.7	56.7 ± 16.4	0.351
Calculated LDL (mg/dL)	129.1 ± 44.4	132.4 ± 48.2	125.2 ± 27.3	132.5 ± 49.1	121.2 ± 34.0	0.366
Creatinine (mg/dL)	0.97 ± 1.04	0.76 ± 0.41	0.78 ± 0.13	0.80 ± 0.34	0.87 ± 0.23	0.492
GFR <60 mL/min/1.73 m^2^	12 (15.8%)	1 (7.1%)	2 (11.1%)	1 (16.7%)	11 (13.3%)	0.307

Values are number (%) or mean ± SD. The *P* values reflect comparison between 4 subtypes. *p* values <0.05 are in bold.

**Table 4:** Patient characteristics categorized by hypertension subtypes in the treated group.

Characteristic	All (*n* = 1,040)	Well-controlled HT (*n* = 312)	HT with white-coat effect (*n* = 242)	Masked uncontrolled HT (*n* = 100)	Sustained HT (*n* = 386)	*p* value
Age (years)	59.0 ± 12.5	58.9 ± 12.5	58.5 ± 12.3	59.3 ± 12.5	59.2 ± 12.6	0.907
Female	618 (59%)	195 (62.5%)	140 (57.9%)	54 (54.0%)	229 (59.3%)	0.441
Diabetes	157 (15.1%)	37 (11.8%)	38 (15.7%)	14 (14.0%)	68 (17.6%)	0.362
Dyslipidemia	558 (53.7%)	169 (54.2%)	131 (54.1%)	52 (52%)	206 (53.4%)	0.854
BMI (kg/m2)	26.6 ± 5.2	26.3 ± 4.5	26.7 ± 4.9	26.7 ± 4.7	26.8 ± 5.9	0.717
Clinic BP						
Systolic BP (mmHg)	143.1 ± 18.1	125.7 ± 9.0	150.8 ± 11.3	127.8 ± 9.1	156.3 ± 14.2	**<0.001**
Diastolic BP (mmHg)	84.1 ± 11.7	76.45 ± 7.6	87.4 ± 9.8	76.7 ± 8.4	90.2 ± 11.7	**<0.001**
Pulse (beats/min)	79.2 ± 12.2	77.61 ± 10.3	80.6 ± 13.3	77.8 ± 12.0	79.9 ± 12.9	**0.012**
Home BP						
Systolic BP (mmHg)	134.4 ± 14.2	122.9 ± 6.9	125.8 ± 6.3	145.5 ± 8.1	147.1 ± 11.3	**<0.001**
Diastolic BP (mmHg)	80.4 ± 8.8	74.6 ± 5.6	76.0 ± 5.6	84.9 ± 6.4	86.6 ± 8.3	**<0.001**
Pulse (beats/min)	74.4 ± 10.1	73.9 ± 8.9	74.1 ± 10.0	75.1 ± 10.9	74.7 ± 10.9	0.599
Laboratory results						
Total cholesterol (mg/dL)	198.2 ± 40.2	196.3 ± 36.9	199.1 ± 42.2	196.0 ± 42.1	200.0 ± 41.2	0.678
Triglyceride (mg/dL)	153.8 ± 86.2	148.0 ± 86.4	157.3 ± 86.4	153.5 ± 72.8	156.6 ± 89.1	0.603
HDL (mg/dL)	53.3 ± 14.8	54.0 ± 13.2	53.6 ± 15.3	52.1 ± 12.5	52.9 ± 16.4	0.731
Calculated LDL (mg/dL)	113.9 ± 37.8	112.3 ± 33.9	113.8 ± 41.1	112.0 ± 39.6	115.9 ± 38.5	0.687
Creatinine (mg/dL)	0.94 ± 0.50	0.89 ± 0.28	0.90 ± 0.32	0.92 ± 0.35	1.02 ± 0.71	0.007
GFR <60 mL/min/1.73 m^2^	256 (28.2%)	76 (27.3%)	54 (26.6%)	23 (26.4%)	101 (30.1%)	0.802

Values are number (%) or mean ± SD. The *p* values reflect comparison between 4 subtypes. *p* values <0.05 are in bold. Abbreviations as Table 1.

**Table 5:** Prevalence of hypertension (HT) subtypes and BP control patterns further classified according to gender and age (≤60 years and >60 years).

	Male		*p* value	Female		*p* value
	Age ≤60 years	Age >60 years		Age ≤60 years	Age >60 years	
Nonmedicated patient (*n* = 144)	53 (100%)	16 (100%)	0.604	55 (100%)	20 (100%)	0.565
Normotension	4 (7.5%)	3 (18.7%)		5 (9.1%)	2 (10.0%)	
White-coat HT	10 (18.9%)	5 (31.2%)		18 (32.7%)	4 (20.0%)	
Masked HT	3 (5.7%)	1 (6.3%)		5 (9.1%)	1 (5.0%)	
Sustained HT	36 (67.9%)	7 (43.8%)		27 (49.1%)	13 (65.0%)	

Treated patient (*n* = 1,040)	212 (100%)	216 (100%)	0.138	329 (100%)	283 (100%)	0.230
Well-controlled HT	64 (30.1%)	55 (25.5%)		95 (28.8%)	101 (35.7%)	
HT with white-coat effect	58 (27.4%)	45 (20.8%)		75 (22.8%)	62 (21.9%)	
Masked uncontrolled HT	19 (9.0%)	27 (12.5%)		27 (8.2%)	27 (9.5%)	
Sustained HT	71 (33.5%)	89 (41.2%)		132 (40.2%)	93 (32.9%)	

Controlled HBP^a^	122 (57.5%)	100 (46.3%)	**0.020**	170 (51.7%)	163 (57.6%)	0.151
Uncontrolled HBP^b^	90 (42.5%)	116 (53.7%)^*∗*^		159 (48.3%)	120 (42.4%)^*∗*^	

Values are number. ^a^Controlled HBP included well-controlled HT and HT with white-coat effect. ^b^Uncontrolled HBP included masked uncontrolled HT and sustained HT in treated patients. ^*∗*^*p* value between male age >60 years and female age >60 years = 0.013.

**Table 6:** Prevalence of hypertension (HT) subtypes and BP control patterns further classified according to the hospital regions of Thailand.

	North	Northeast	East	Central	South	*p* value
Nonmedicated patient (*n* = 144)	7 (100%)	12 (100%)	80 (100%)	34 (100%)	11 (100%)	0.056
Normotension	2 (28.5%)	2 (16.7%)	6 (7.5%)	3 (8.8%)	1 (9.1%)	
White-coat HT	1 (14.3%)	4 (33.3%)	23 (28.8%)	7 (20.6%)	2 (18.2%)	
Masked HT	0	1 (8.4%)	5 (6.2%)	1 (2.9%)	3 (27.3%)	
Sustained HT	4 (57.1%)	5 (41.6%)	46 (57.5%)	23 (67.7%)	5 (45.4%)	

Treated patient (*n* = 1,040)	240 (100%)	264 (100%)	150 (100%)	218 (100%)	168 (100%)	0.197
Well-controlled HT	82 (34.2%)	90 (34.1%)	32 (21.3%)	57 (26.1%)	51 (30.4%)	
HT with white-coat effect	49 (20.4%)	61 (23.1%)	38 (25.3%)	59 (27.1%)	35 (20.8%)	
Masked uncontrolled HT	25 (10.4%)	22 (8.3%)	15 (10.1%)	25 (11.5%)	13 (7.7%)	
Sustained HT	84 (35.0%)	91 (34.5%)	65 (43.3%)	77 (35.3%)	69 (41.1%)	

Controlled HBP^a^	131 (54.6%)	151 (57.2%)	70 (46.7%)	116 (53.2%)	86 (51.2%)	0.317
Uncontrolled HBP^b^	109 (45.4%)	113 (42.8%)	80 (53.3%)	102 (46.8%)	82 (48.8%)	

Values are number. The *p* values reflect the comparison between 5 regions. ^a^Controlled HBP included well-controlled HT and HT with white-coat effect. ^b^Uncontrolled HBP included masked uncontrolled HT and sustained HT in treated patients.

## Data Availability

The related data used to support the results of this study are available from the corresponding author upon request.

## Supplementary Materials

Supplementary Materials Supplementary list of all 46 participating sites. Supplementary Figure 1: specification of the oscillometric home blood pressure monitoring device used in the study (Uright model TD-3128, TaiDoc Technology Corporation, Taiwan). Supplementary Figure 2: patient enrollment flow chart of the study. Supplementary Table 1: clinical validation of home blood pressure device according to British Hypertension Society grading criteria.
