# Supplementary material for: Hypertension Subtypes among Thai Hypertensives: An Analysis of Telehealth-Assisted Instrument in Home Blood Pressure Monitoring Nationwide Pilot Project
Source: Int J Hypertens. 2020 Apr 9;2020:3261408. doi: 10.1155/2020/3261408 (PMC7171656; doi:10.1155/2020/3261408)
Supplement: Supplementary Materials — Supplementary list of all 46 participating sites. Supplementary Figure 1: specification of the oscillometric home blood pressure monitoring device used in the study (Uright model TD-3128, TaiDoc Technology Corporation, Taiwan). Supplementary Figure 2: patient enrollment flow chart of the study. Supplementary Table 1: clinical validation of home blood pressure device according to British Hypertension Society grading criteria. [file 3261408.f1.docx]

**Supplementary List of all 46 participating sites**

Vajira hospital; Bangkok (Peth Rod-aree), Prapokklao hospital; Chanthaburi (Wanna Chamjamrat), Uttaradit hospital; Uttaradit (Phatcharee Phengsupun), Maemoh hospital ; Lampang (Atitaya Churdchom), Mae On hospital; Chiang Mai (Linda Inphom), Banhong hospital; Lamphun (Pattaraporn Wongyai), Chiangkong Prince Crown hospital; Chaing Rai (Sukanya Sriprapaporn), Laplae hospital; Uttaradit (Chonlachab Jundoung), Aoluk hospital; Krabi (Chanisara Chaisiri), Pakpayun hospital; Phatthalung (Pimprapai Buakeaw), Khokcharoen hospital; Lopburi (Nuntawan Khwansuk), Chaiyo hospital; Angthong (Kunyarat Thongsod), Doembangnangbuat hospital; Suphan buri (Orachorn Panich), Phanomsarakham hospital; Chachoengsao (Dungmanee Wiyathus), Wapipathum hospital; Maha Sarakham (Benjaporn Intakornudom), Nawa hospital; Nakhon Phanom (Areerath Phaengyod), Dokkhamtai hospital; Phayao, Tha Wang Pha hospital; Nan, Song hospital; Phrae, Bang Pahan hospital; Ayutthaya, Mae Ra Mard hospital; Tak, Sawang Arom hospital; Uthai Thani, Thalang hospital; Phuket, Ban Na San hospital; Surat Thani, Pathio hospital; Chumphon, Bannang Sata hospital; Yala, Prajan Takam hospital; Prachinburi, Angthong hospital; Angthong, Bo rai hospital; Trat, Ko Sichang hospital; Chonburi, Bang Bo hospital; Samut Prakan, and King Chulalongkorn Memorial hospital; Bangkok

**
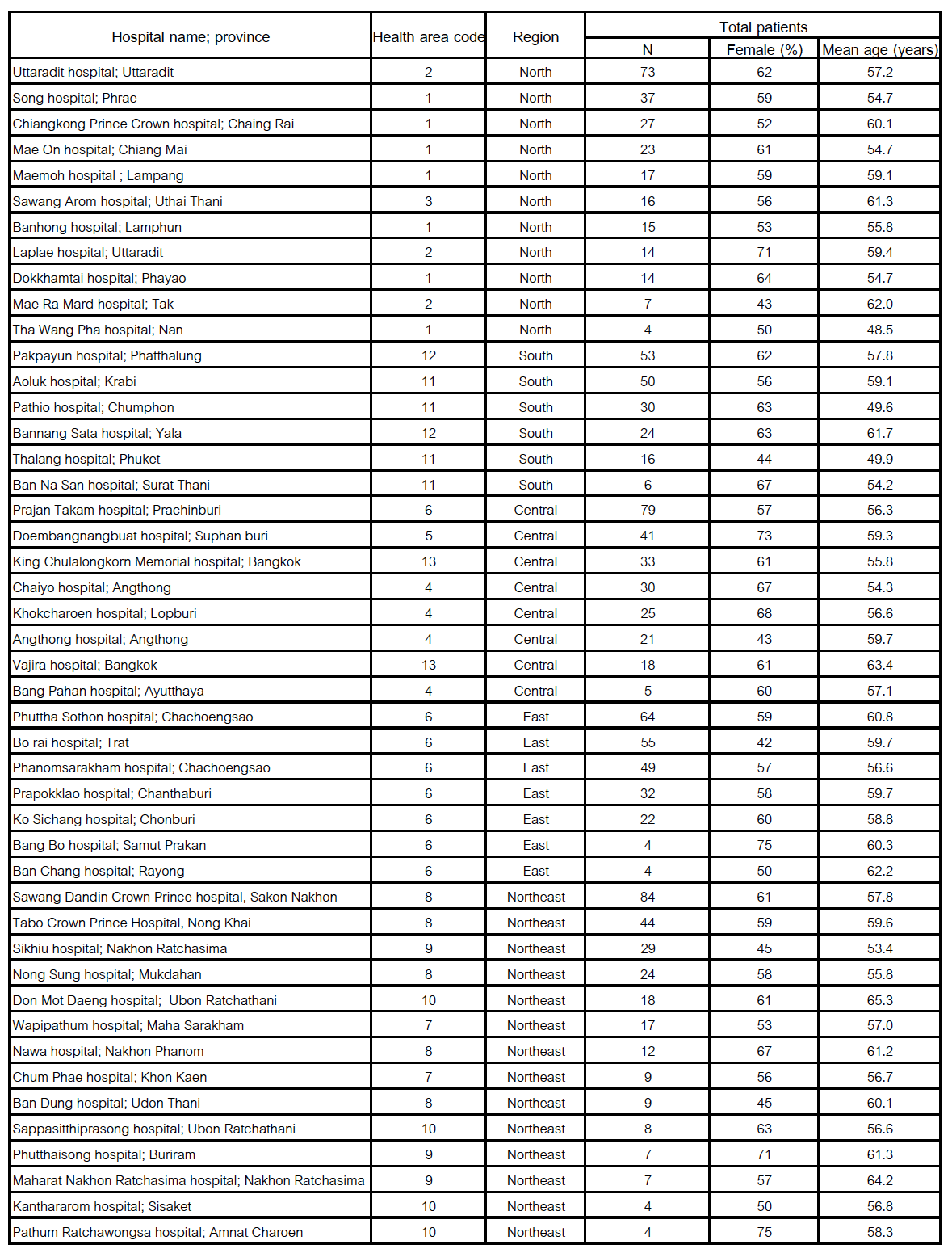
**

**Supplementary Figure 1:** Specification of the oscillometric home blood pressure monitoring device used in the study (Uright model TD-3128, TaiDoc Technology Corporation, Taiwan)


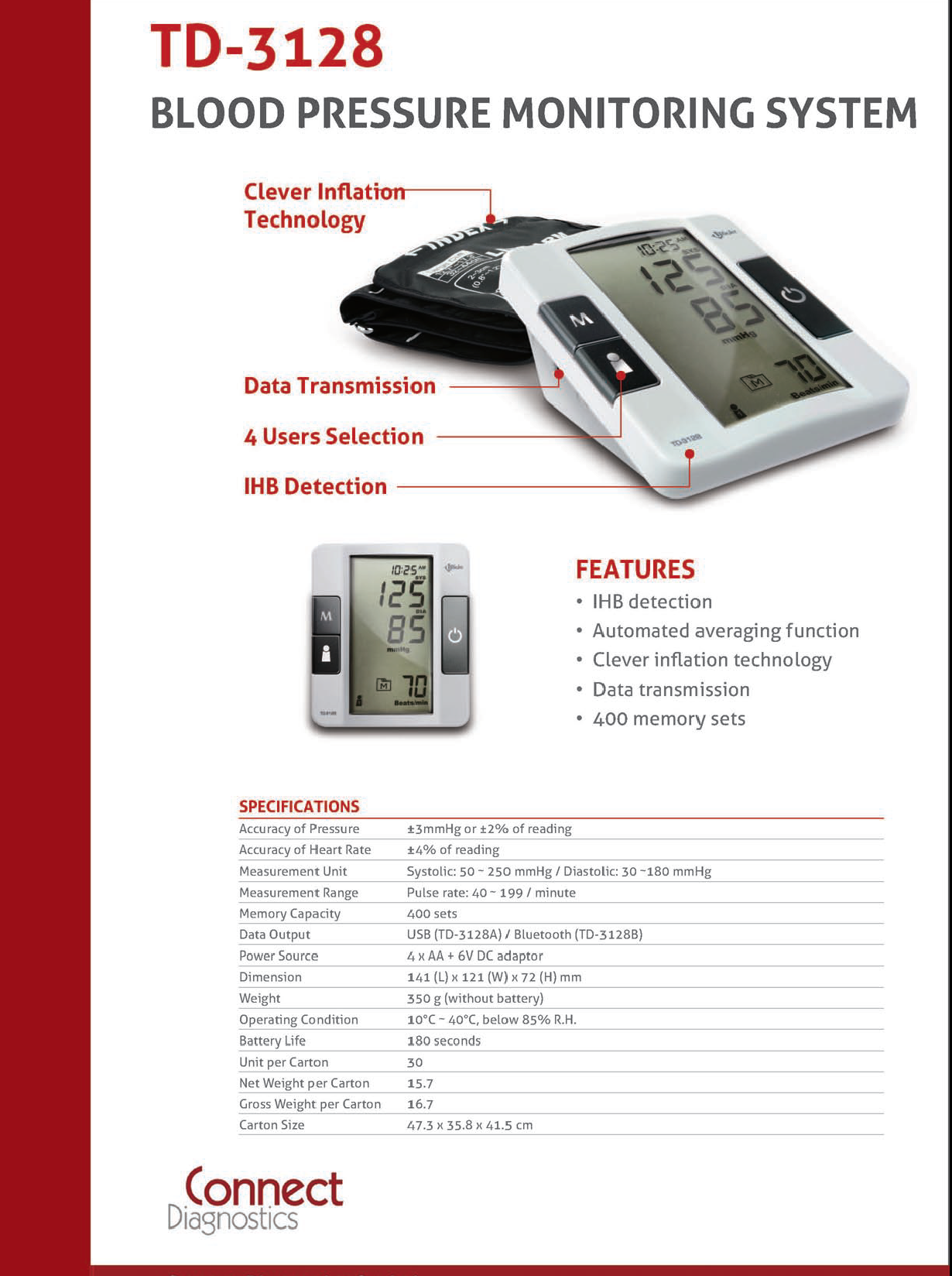


**Supplementary Table 1:** Clinical validation of home blood pressure device (Uright TD-3128 devices, TaiDoc Technology Corporation) according to British Hypertension Society grading criteria.

| **Systolic blood pressure** | | | |
| --- | --- | --- | --- |
| < 5mmHg | < 10 mmHg | < 15 mmHg | Total |
| N = 597 | N = 696 | N = 714 | N = 727 |
| 82.1% | 95.7% | 98.2% | **Grade A** |
| **Diastolic blood pressure** | | | |
| < 5mmHg | < 10 mmHg | < 15 mmHg | Total |
| N = 582 | N = 677 | N = 703 | N = 727 |
| 80.1% | 93.1% | 96.7% | **Grade A** |

**Supplementary Figure 2**: Patient enrollment flow chart

**n = 1,288**

**Failed clinical validation between clinic and home blood pressure readings (n=38)**

**n = 1,250**

**n = 1,184**

**Incomplete clinical characteristics or blood pressure data (n = 66)**
